# Postoperative Eyelid Pyogenic Granuloma: A Systematic Review of Clinical Features, Surgical Associations, and Management

**DOI:** 10.7759/cureus.108239

**Published:** 2026-05-04

**Authors:** Imogen Cheung, David Cheung

**Affiliations:** 1 Ophthalmology, Leicester University, Leicester, GBR; 2 Ophthalmology, Birmingham and Midland Eye Centre, Birmingham, GBR

**Keywords:** eyelid, lobular capillary haemangioma, oculoplastic surgery, postoperative complication, pyogenic granuloma, wound healing

## Abstract

Pyogenic granuloma (lobular capillary haemangioma) is a benign vascular lesion that may arise following trauma, inflammation, or surgery. In oculoplastic practice, it represents a recognised but likely underreported postoperative complication. We present a case of recurrent pyogenic granuloma arising from a posterior lamellar defect following eyelid reconstruction using a Hughes flap, highlighting key clinical features and management considerations. A systematic review of the literature was also performed to contextualise this case, identifying 14 studies comprising 498 cases. Lesions typically presented within weeks of surgery as rapidly growing, friable, pedunculated masses arising at sites of surgical disruption. While topical therapies such as corticosteroids and timolol may be effective in selected cases, surgical excision remains the most reliable treatment and allows histological confirmation. This case and systematic review support the theory that postoperative pyogenic granuloma represents a manifestation of dysregulated wound healing driven by aberrant angiogenesis at sites of epithelial disruption.

## Introduction

Pyogenic granuloma is a vascular inflammatory lesion often involving the skin or mucosa [1,2]. These lesions typically arise suddenly, resulting in a smooth-surfaced, thin-walled, red, erythematous, polyp-like growth which can bleed spontaneously or from light trauma, e.g., touching or wiping. The term “pyogenic granuloma” was first introduced by Hartzell in 1904 to describe a vascular lesion thought to represent an infectious granulomatous process [3]. There has been confusion about this lesion entity over history, partly due to its multiple names, including Croker and Hartzell disease, fibroangioma, polypoid capillary haemangioma, telangiectatic granuloma, and benign pedunculated granuloma [4]. Early ophthalmic descriptions were limited to isolated case reports, with the first comprehensive series of ocular adnexal pyogenic granulomas reported by Ferry in 1989 [5]. Pyogenic granuloma is thought to arise from a disordered vasoproliferative inflammatory response within granulation tissue, resulting in fibroblast proliferation and capillaries that often radiate in lobules; hence the alternative name of lobular capillary haemangioma [4,6]. Despite its name, it is neither pyogenic nor granulomatous but is now thought to be a lobular capillary haemangioma arising from dysregulated wound healing [7]. It is often associated with trauma, inflammation, and surgical intervention, but can arise spontaneously. In ophthalmology, pyogenic granuloma most frequently involves the cornea [8], conjunctiva [9], and eyelids and may arise following minor trauma or surgery. Within oculoplastic practice, it is a recognised complication of chalazia [10] and of many surgical procedures such as chalazion curettage [7,11], blepharoplasty [12], ptosis repair [13,14], reconstructive surgery [15,16], following lacrimal bypass surgery [17], and punctal [18] and intracanalicular plug use [19,20]. Its rapid growth and vascular appearance can be clinically striking and may raise concern for more serious pathology.

The literature remains limited and fragmented, consisting predominantly of case reports and small case series. Consequently, there is no consolidated evidence base addressing postoperative eyelid pyogenic granuloma. This study aims to review the literature systematically to characterise its clinical presentation, associated surgical factors, and management outcomes.

## Case presentation

**Figure 1:**
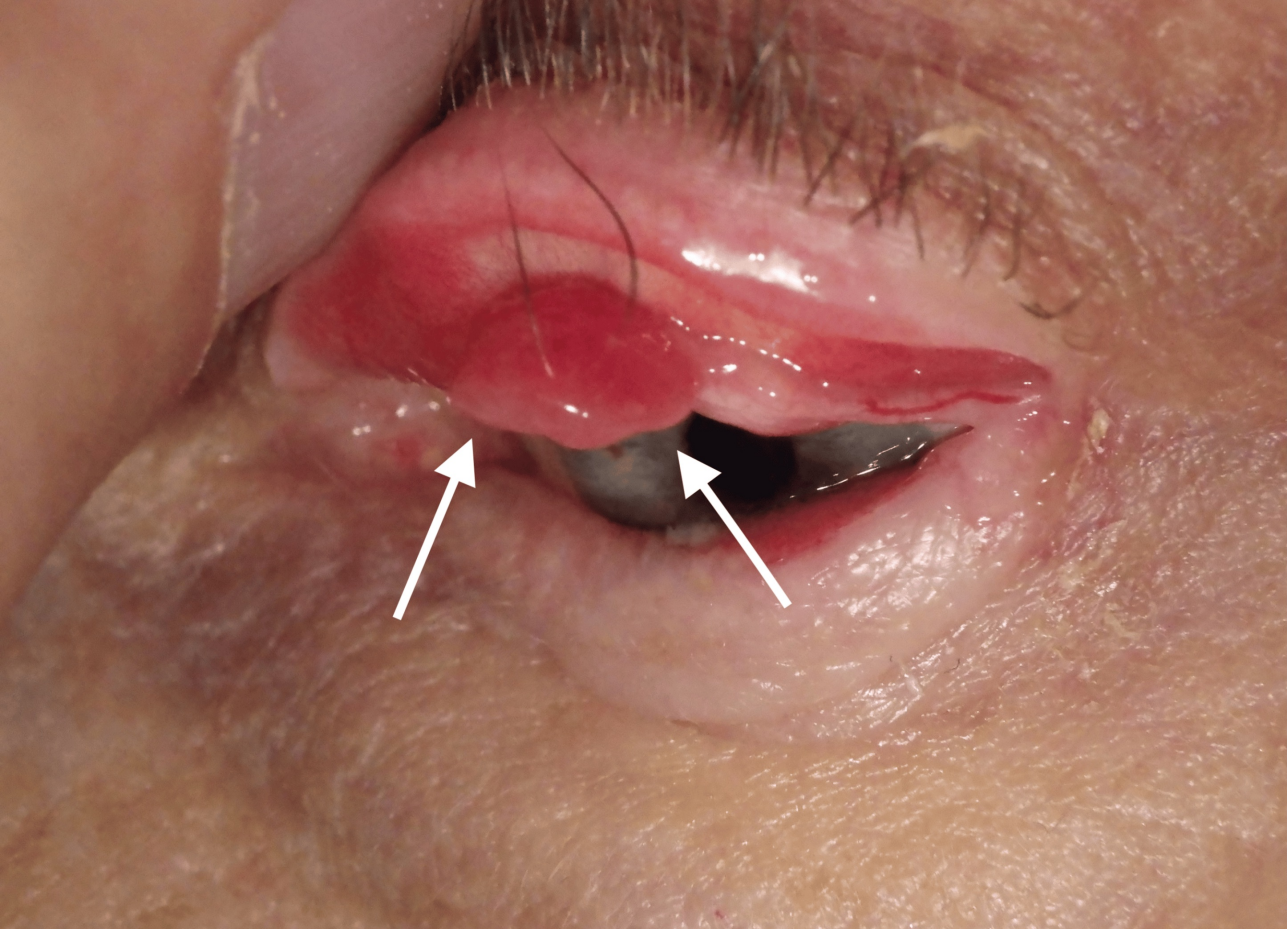
Clinical photograph demonstrating a pedunculated, vascular lesion arising from the tarsal conjunctiva consistent with pyogenic granuloma following eyelid reconstruction. The 12mm wide lesion appears erythematous, friable, and lobulated, typical of a lobular capillary haemangioma.

A 75-year-old asthmatic lady with a known history of meibomian gland dysfunction and previous chalazia presented with a left lower lid classical 15 mm wide noduloulcerative lesion affecting the eyelid margin consistent with basal cell carcinoma. After thorough Montgomery-compliant consent [21], she underwent a full-thickness excisional biopsy of the lesion with a 3 mm margin. Fast paraffin histology showed complete clearance of a nodular BCC. Her reconstruction consisted of a Hughes flap [22] and a left postauricular full-thickness skin graft with division of the Hughes flap two weeks later. However, three months later, she anxiously presented again with left eye serosanguinous discharge and a three-day history of left upper eyelid swelling. Upper lid eversion showed a pedunculated red mass arising near the medial inferior corner of the tarsal plate defect from the previous Hughes flap tarsal flap donor site, consistent with a pyogenic granuloma (Figure 1). 

Atypically, our patient’s lesion had two eyelashes entrapped within its core, which were easily lifted out of the lesion with forceps in the clinic, providing immediate relief. She was started on a course of topical steroids, but there was no improvement after two weeks. Timolol drops were not tried in view of her respiratory history. Excisional biopsy of the lesion was therefore performed under local anaesthetic. Histology was consistent with pyogenic granuloma/lobular capillary haemangioma. A total of 18 months later, the patient presented again with a smaller, similar lesion arising from the medial aspect of the tarsal plate defect of her previous Hughes flap (the lesion was so shallow that it was difficult to capture with photography). She underwent further excisional biopsy of this new lesion, which again was consistent with pyogenic granuloma/lobular capillary haemangioma. She remains recurrence-free following 18 months of follow-up (unfortunately, the histology lab was unable to reproduce the original histology slides for this publication).

## Discussion

**Figure 2:**
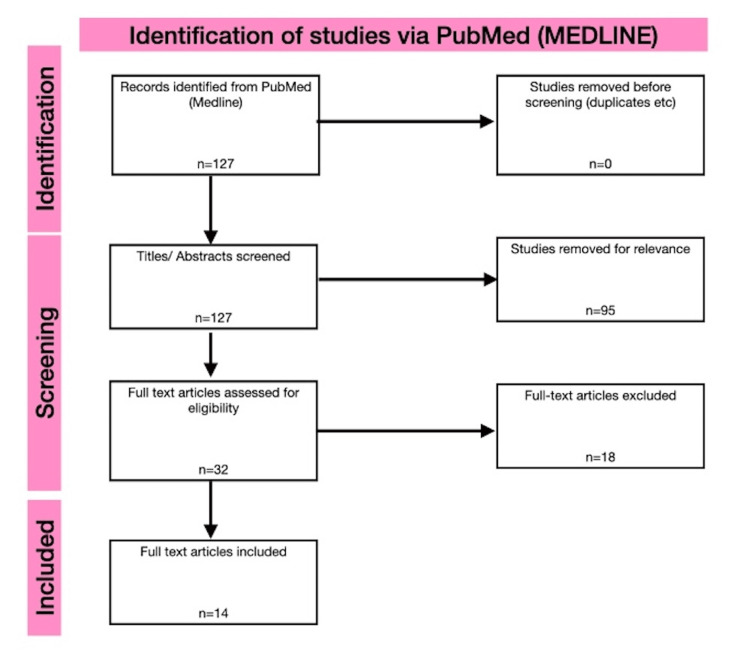
PRISMA flow diagram illustrating study selection process for inclusion in the systematic review.

A systematic review was conducted in accordance with PRISMA guidelines [23]. No formal protocol registration was performed. A literature search of PubMed/MEDLINE was performed up to March 2026 using the following terms: (“pyogenic granuloma” OR “lobular capillary haemangioma”) AND (eyelid OR blepharoplasty OR “eyelid surgery” OR oculoplastic OR chalazion OR ptosis). Reference lists of included articles were also screened. Inclusion criteria included eyelid pyogenic granuloma, post-surgical cases, human studies, English-language publications, case reports, case series, and observational studies. Exclusion criteria consisted of non-eyelid lesions, non-surgical cases, reviews without primary data, and animal studies. Titles and abstracts were screened, followed by full-text review. Extracted data included demographics, procedure type, time to onset, clinical features, treatment, and outcomes. Given the rarity of postoperative eyelid pyogenic granuloma, inclusion of case reports and small series was necessary to allow meaningful results. Spontaneous/ idiopathic pyogenic granuloma cases were excluded. The initial PubMed/MEDLINE database search identified 127 articles ranging from 1964 to 2026. After removal of duplicates and screening of titles and abstracts, 32 full-text articles were assessed for eligibility. Of these, 14 studies met the inclusion criteria. There were multiple studies relating to pyogenic granuloma formation secondary to punctal and intracanalicular plug insertion. These were not included (Figure 2).

The included studies comprised a combination of case reports and small case series, reflecting the rarity of postoperative eyelid pyogenic granuloma. In total, 498 patients were identified across 14 studies (Table 1). We looked at what types of surgical procedures were associated with postoperative eyelid pyogenic granuloma formation, their clinical presentation, management strategies, and recurrence rates.

**Table 1:** Studies of postoperative eyelid pyogenic granuloma. 'Incidence' refers to the proportion of pyogenic granuloma occurring within the reported surgical cohort where applicable.

Study	Year	Study Type	No. of PG cases	Procedure / Context	Sex	Mean Age (Range)	PG rate associated with that procedure	PG recurrence rate	Predisposing Factor	Time to Onset	Site	Management
Retrospective studies of all patients presenting with PG to their practice												
Ferry AP [4]	1989	Case series (n = 100)	100	Mixed (chalazion, surgery, trauma)	Equal	34 (2–91)	Not applicable	3%	Chalazion related, surgical wound interface	Variable	Tarsal Conjunctiva / Skin	Excision
Jordan DR et al. [7]	2001	Case series (n = 16)	16	Oculoplastic procedures	Equal	50 (8–82)	Not applicable	Low	Surgery, trauma	Weeks	Tarsal Conjunctiva / skin	Largely Excision, few cases treated with topical steroids but ineffective
Al-Towerki AA [11]	1996	Case series (n = 110)	110	Predominantly oculoplastic surgery	Equal	39.4 (2–80)	Not applicable	~8%	Entropion surgery, others	Weeks	Conjunctiva / wound	Excision majority
Wu D et al. [24]	2017	Case series (n = 46)	46	Surgery, trauma, chalazion	Male predominance	23.5 (2–68)	Not applicable	Not reported	Surgery (47.8%), chalazion	~2.5 months	Bulbar / tarsal conjunctiva	Excision
Retrospective studies of specific techniques												
Pham et al. [25]	2022	Case series (n = 6)	1	Free tarsoconjunctival graft with skin muscle flap	Not reported	61.3 (36–91)	16.7% (1 of 6)	0	Surgical Wound interface	Weeks	Tarsal Conjunctival	Excision
Hishmi et al. [15]	2016	Case series (n = 45)	2	Hughes flap reconstruction	Not reported	75 (40–96)	4% (2 of 45)	0	Surgical Wound interface	Weeks	Tarsal Conjunctiva	Excision
Mullins et al. [26]	1997	Case series (n = 400)	8	Transconjunctival blepharoplasty	Not reported	Not reported	~2% (8 of 400)	0	Surgical Wound interface	Weeks–months	Tarsal Conjunctiva	Excision
Bair et al. [14]	2025	Case series (n = 105)	1	Sutureless MMCR	Female predominant	67	~1% (1 of 105)	0	Surgical Wound interface	Weeks	Tarsal Conjunctiva	Excision
McNutt et al. [16]	2015	Case series (n = 41)	10	Rotational flap repair without posterior lamellar graft or flap	Not reported	74	24.4 (10 of 41)	0	Surgical Wound interface /Suture related	Weeks/ months	Conjunctiva	Topical Steroids/ Excision
Gower et al. [27]	2011	Case series (n = 1881)	198	Trachamatous Trichiasis lid rotation surgery	Female predominant	Not reported (majority >50)	10.5% (198 of 1881)	Not reported	Surgical Wound interface	Weeks	Tarsal Conjunctival	Excision
Perry et al. [28]	2013	Case series (n = 18)	2	Lateral cantholysis for 14-21mm horizontal defect reconstruction	Largely equal	73 (45-94)	11% (2 of 18)	0	Surgical Wound interface	Week/ months	Tarsal conjunctival	Excision
Philips et al. [29]	2019	Case series (n = 9)	1	V shaped lateral canthoplasty for floppy eye lid syndrome	Male predominance	65	11% (1 of 9)	0	Surgical Wound interface	Weeks/ months	Lateral canthus	Excision
Lessa et al. [30]	2019	Case series (n = 136)	2	Non sutured transconjunctival lower blepharoplasty	Not reported	Not reported	1.47 (2 of 136)	0	Surgical Wound interface	Weeks/ months	Conjunctival	Excision
Single case reports												
Soll SM et al. [12]	1993	Case report (n = 1)	1	Transconjunctival blepharoplasty	Not reported	Not reported	Not applicable	0	Surgical Wound interface	Weeks	Tarsal Conjunctiva	Excision

Pyogenic granuloma was most frequently reported following chalazion incision and curettage through the same tarsal plate incision, e.g., 42 of 100 cases in Ferry’s study [5]. Less commonly, in other types of oculoplastic surgery, including blepharoplasty, ptosis repair procedures, and eyelid reconstruction surgery. The interval between surgery and development of pyogenic granuloma ranged from approximately 1 to 6 weeks, with most cases presenting within the first 2-4 weeks postoperatively. Lesions were typically described as rapidly growing, red or reddish purple, and often pedunculated or sessile. During surgery, they were described as friable, with a high tendency to bleed. Postoperative eyelid pyogenic granuloma commonly arises from the tarsal conjunctiva and eyelid margin, usually from the site of a previous surgical incision.

Management approaches varied across studies and included topical corticosteroids, intralesional steroid injections, topical timolol, surgical excision or curettage, and occasional use of cauterisation. Topical therapy was often effective in smaller lesions, while larger or persistent lesions required surgical removal. Some authors strongly advocated excision, particularly in patients who have undergone reconstruction surgery for sinister pathology previously. Outcomes were generally favourable across all treatment modalities. Recurrence was uncommon but reported in a small number of cases, particularly where initial treatment was conservative (Table 1). 

In clinical practice, postoperative eyelid pyogenic granuloma is often first identified during routine follow-up or prompted by patient concern regarding a rapidly growing lesion. A typical presentation, as seen in our case report, is the development of a small, red, fleshy lesion along a conjunctival or incision site approximately two to three weeks following chalazion surgery or blepharoplasty. This timing is consistent with the proliferative phase of wound healing and supports the theory that pyogenic granuloma represents an exaggerated angiogenic response rather than a true neoplastic process [7]. A pyogenic granuloma can be understood as a disturbance of normal wound healing, in which the tightly regulated balance between angiogenic stimulators and inhibitors becomes disrupted. During physiological repair, capillary proliferation and granulation tissue formation are transient processes that subside once epithelial closure is achieved. However, in the presence of ongoing tissue irritation or inflammation, this balance is lost, leading to excessive endothelial proliferation and capillary sprouting. The resulting vascular tissue may protrude through the wound surface, mechanically preventing epithelial closure and perpetuating a self-sustaining cycle of angiogenesis and incomplete healing [7]. This conceptual model is supported by clinical and histopathological data. In a series of 46 patients with medically uncontrolled conjunctival pyogenic granulomas, two distinct histological patterns were identified: an inflammatory subtype characterised by prominent capillary proliferation and dense inflammatory cell infiltration, and a fibrous subtype with relatively sparse vascularity and increased stromal fibrosis [24]. The inflammatory pattern was more commonly associated with prior surgery or trauma, whereas fibrous lesions were frequently linked to chronic chalazion. These findings suggest that pyogenic granuloma represents a spectrum of dysregulated wound healing responses, with the relative balance of angiogenesis and inflammation determining the clinical and histological phenotype [24]. Although benign, these lesions can sometimes cause disproportionate anxiety due to their rapid growth and bleeding tendency, sometimes resulting in a serosanguinous discharge or even frank bloody tears/haemolacria [31], whilst being present completely asymptomatically in almost half of cases [11]. In some cases, they may be confused with infection, recurrent chalazion, or neoplastic lesions [11,32,33].

It is notable that postoperative pyogenic granuloma lesions typically arose through the point of breach of the eyelid previously. For example, in patients who developed pyogenic granuloma associated with chalazion, the lesion would typically arise from either the tarsal plate aspect where the chalazion had previously ruptured through or where previous incision and curettage drainage had been performed [5]. In patients who suffered spontaneous rupture of a chalazion through the skin, their pyogenic granuloma would arise as a cutaneous lesion through the previous point of cutaneous rupture. Our illustrative case where our patient suffered two pyogenic granulomas arising from an area where the posterior eyelid surface was devoid of tarsus, is consistent with these findings and suggests that the point of eruption of pyogenic granuloma coincides with an area of weakness devoid of normal epithelial tissue. This observation is consistent with the theory that pyogenic granuloma arises from dysregulated wound healing in which delayed or incomplete re-epithelialisation permits persistent granulation tissue proliferation and excessive angiogenesis [11]. Such lesions are frequently associated with ulcerated epithelial surfaces and ongoing local irritation, further perpetuating the process [7,27,30,34]. Interestingly, a markedly higher incidence of pyogenic granuloma (24.4%) has been reported following rotational flap reconstruction without posterior lamellar repair [27]. In this series, lesions arose at the flap-tissue interface and were attributed in part to the use of gut sutures. This raises the possibility that perhaps some reported lesions may represent an exaggerated foreign body or suture-related granulomatous response rather than a classical pyogenic granuloma/lobular capillary haemangioma, particularly given the substantially higher incidence compared to other oculoplastic series. There is, therefore, some degree of diagnostic doubt in this study and perhaps others as to what their observed lesions were, since histological confirmatory analysis was not performed. It, therefore, remains difficult to draw conclusions as to which operations and, therefore, which causative factors are associated with pyogenic granuloma formation. In Ferry’s prospective study of 100 pyogenic granuloma cases, for example [5], 42 cases arose from previous cases of chalazion. However, this does not necessarily mean that incision and curettage as an operation are more likely to cause a pyogenic granuloma but may be related to incision and curettage being the most frequently performed operation in his practice. Nevertheless, these findings reinforce the importance of the local wound environment and inflammatory stimuli in driving reactive vascular proliferations. Further support for a wound-healing-driven mechanism is provided by studies of posterior lamellar surgery [15,26]. In a large series of 105 eyelids undergoing sutureless Müller’s muscle-conjunctival resection, pyogenic granuloma occurred in one case following conjunctival resection [14]. The authors note that tissue healing in this context relies on natural adhesion and re-epithelialisation, suggesting that disruption of conjunctival integrity alone may be sufficient to trigger aberrant granulation tissue formation [14]. Large cohort data further support a surgically mediated mechanism. Gower et al. reported a 10.5% incidence of pyogenic granuloma following trichiasis surgery, with significant variation between surgeons, suggesting a strong influence of surgical technique [27]. The authors proposed that pyogenic granuloma formation may arise from local irritation at the wound edge, including rough tarsal surfaces or retained suture material, reinforcing the concept that dysregulated wound healing and mechanical factors play a central role. In addition to mechanical factors, biochemical irritation may also contribute to lesion formation. Meibomian gland secretions have been implicated as a potential pro-inflammatory stimulus, particularly in chalazion-associated cases, where lipid extravasation may promote persistent local inflammation and granulation tissue formation [11,25]. It is interesting that both our pyogenic granuloma lesions arose on the inferior edge of our Hughes flap, i.e., transecting through meibomian glands, reinforcing this theory. Other authors who studied pyogenic granuloma formation in other types of ophthalmic surgery or accidental trauma found that a breach of the conjunctiva at the same site as the pyogenic granuloma formation was a common predisposing factor [5,11,34]. However, in some cases, even minimal repetitive conjunctival stress was associated with pyogenic granuloma formation in some, e.g., contact lens wear and dry eye [11]. Whether the surface of eruption in tarsal plate pyogenic granulomas is related to the location of irritation and, therefore, the site of dysregulated angiogenesis or whether it is due simply to the tarsal plate being inherently weaker at this point, e.g., due to previous surgery or rupture, remains unknown. We suspect that it may be a mixture of both these factors.

The incidence of recurrent postoperative eyelid pyogenic granuloma is low for idiopathic spontaneous lesions treated with surgery, but is surprisingly higher in cases associated with previous surgery, at 3-8% in some studies [5,11]. Given the theories regarding how dysregulated angiogenesis can arise in areas of increased inflammation and irritation, it is surprising that pyogenic granuloma lesions do not arise more frequently following upper eyelid surgery where a posterior lamellar defect is allowed to heal laissez-faire [15,25], e.g., tarsal plate grafts and Hughes flaps as in our case.

Based on literature and clinical experience, a pragmatic approach for small lesions is a trial of topical steroid and topical timolol [9,35,36], which can be instigated in the clinic. Evidence-based practice remains challenging due to the predominance of small case series, lack of histological confirmation in some reports, and heterogeneity in outcome reporting. For larger or persistent lesions, other authors have recommended intralesional steroids or surgical excision [7,11]. Unfortunately, eyelid pyogenic granuloma can cause diagnostic uncertainty and patient anxiety by masquerading as more sinister pathology, often presenting in patients who have undergone previous oculoplastic surgery for eyelid cancers [32,33]. Conversely, sinister pathology can present to look like and therefore be misdiagnosed as pyogenic granuloma lesions [37,38], and rarely have past authors described spontaneous pyogenic granuloma formation in patients with underlying conjunctival epithelial neoplasia [39]. For these reasons, we recommend that surgical excision be the preferred option, as it can be performed quickly, sometimes in the clinic under topical anaesthesia only, with rapid symptom resolution and reassuring histological confirmation of the suspected diagnosis.

## Conclusions

Postoperative eyelid pyogenic granuloma is an uncommon but likely underreported complication of oculoplastic surgery that typically presents rapidly and may cause both patient anxiety and diagnostic uncertainty. Predisposing factors and optimal management remain difficult to define due to small study sizes and the frequent absence of histological confirmation. However, the consistent observation that these lesions arise at sites of surgical disruption supports the theory that they result from aberrant angiogenesis within a dysregulated wound healing response. While topical therapies such as corticosteroids and timolol may be effective in selected cases, surgical excision, often performed under local anaesthesia, appears to provide the most reliable treatment, with the added benefit of histological confirmation and rapid reassurance for both patient and clinician.
